# Editorial: Orphan GPCRs As Emerging Drug Targets

**DOI:** 10.3389/fphar.2015.00295

**Published:** 2015-12-15

**Authors:** Ye Fang, Terry Kenakin, Changlu Liu

**Affiliations:** ^1^Biochemical Technologies, Science and Technology Division, Corning IncorporatedCorning, NY, USA; ^2^Department of Pharmacology, University of North Carolina School of MedicineChapel Hill, NC, USA; ^3^Neuroscience Therapeutic Area, Janssen Global Services, LLCSan Diego, CA, USA

**Keywords:** G protein-coupled receptor, orphan GPCR, drug discovery, review

G protein coupled-receptors (GPCRs) have been proven to be the most successful class of druggable targets in the human genome. In fact, 30–50% of marketed drugs are estimated to exert their clinical effects via GPCRs. Furthermore, the pace of GPCR-targeted new molecular entities (NMEs) approved by the US Food and Drug Administration (FDA) in the recent years still remains to a level near its historical average, with five in 2010, five in 2011, seven in 2012, six in 2013, and eight in 2014. Out of total 219 NMEs approved by the FDA from 2005 to 2014, 54 (25%) target GPCRs.

GPCRs are and continue to be one of the most popular target classes for drug discovery. This is in part due to the involvement of GPCRs in regulating a wide range of human physiological and pathophysiological processes, and in part due to the fact that current GPCR drugs only target ~80 known receptors, about 10% of all GPCRs encoded in the human genome. Efforts in elucidating the structure, biology, and therapeutic effects of remaining GPCRs, in particular ~140 orphan receptors whose natural ligands are mostly unknown, shall continue to fuel the interest in GPCR based drug discovery. Given the tremendous progresses in exploiting the chemical and biological spaces of many orphan GPCRs, we thought to promote a Research Topic with the contribution of top-leading scientists from both academic and industrial laboratories who have been active in the field of translational research of orphan GPCRs.

Ichimura et al. review the molecular pharmacology, biology, and potential clinical applications of free fatty acid receptors (FFARs). FFARs consist of four family members. Short-chain fatty acids that contain fewer than six carbons activate FFAR3 and FFAR2, while fatty acids that contain more than six carbons activate both FFAR1 and FFAR4. Fatty acids are both energy source and signaling molecules that regulate energy metabolism and other physiological processes. FFARs represent promising targets for developing innovative drugs to treat metabolic disorders associated with dysfunction of energy balance, such as obesity, type 2 diabetes, and inflammatory bowel disease. Although the FFAR1 agonist fasiglifam phase III trial for the treatment of type 2 diabetes discontinued due to liver toxicity, several clinical trials targeting FFARs are still ongoing.

Thurmond reviews the discovery, molecular pharmacology, function, and clinical data of histamine H_4_ receptor. The discovery of four histamine receptors is a result of almost 100 years long endless efforts. Both H_1_ and H_2_ receptors were discovered based on clinical pharmacology and ligand binding, while the H_3_ receptor was first proposed based on pharmacology and later confirmed using a reverse pharmacology approach. In contrast, H_4_ receptor was discovered with an orphan GPCR gene sequence followed by pharmacology characterization. Literature mining of histamine activities unrelated to the other three histamine receptors suggests that H_4_ ligands may be useful to treat asthma and chronic pruritus associated with conditions such as atopic dermatitis. Animal models suggested a role for the H_4_ receptor in mediating pruritic responses, and lung function and inflammation. The H_4_ antagonist JNJ 39758979 has recently been found to have efficacy in preclinical models of pruritus, dermatitis, asthma, and arthritis. Several other H_4_ antagonists have also been entered into clinical trials for these indications.

Sun and Liu review the de-orphanization, molecular pharmacology, and biology of EBI2 (GPR183). 7α,25-dihydroxyxcholesterol, an oxysterol, was identified to be the likely physiological ligand of EBI2. EBI2 has been implicated in regulating migration, activation, and functions of B cells, T cells, dendritic cells, monocytes/macrophages, and astrocytes. EBI2 may represent a promising target for therapeutic interventions to treat diseases associated with dysregulation of the synthesis or functions of oxysterols.

Divorty et al. review the molecular pharmacology, signaling, function, and therapeutic potential of GPR35. Several endogenous molecules including kynurenic acid, cGMP, DHICA, reverse T3, and CXCL7 can activate GPR35; however, none has yet been confirmed to be the true endogenous agonist for the receptor. Both endogenous and synthetic agonists identified were found to display biased agonism and species selectivity. Genome-wide association and functional studies suggest that GPR35 is associated with a wide range of diseases, such as inflammatory bowel disease, type 2 diabetes, coronary artery disease, heart failure and hypoxia, inflammation, pain transduction, and synaptic transmission.

Shore and Reggio review the molecular pharmacology, expression patterns, potential clinical applications of both GPR35 and GPR55. The authors augment that GPR35 is the CXCR8, since it appears bind to the chemokine CXCL17 with nanomolar affinity. GPR35/CXCR8 has been implicated in numerous pathologies involving the gastrointestinal tract, adrenal glands, lung, uterus, liver, immune system, bladder, kidney, bone, central nervous system, and cardiovascular system. On the other hand, GPR55 has been recently deorphanized to be a receptor for lysophophatidylinositol. Other GPR55 ligands identified so far are neither cannabinoids nor bind to the cannabinoid CB1 and CB2 receptors. GPR55 has been implicated in three therapeutic areas, including the regulation of energy intake and expenditure, resorption of bone, and agonist pro-carcinogensis.

Smith reviews the expression patterns, molecular pharmacology, and therapeutic potentials of GPR37 and GPR37L1, the two endothelin B receptor-like receptors. Head activator is the most likely physiological agonist for GPR37, while the neuropeptides prosaposin and prosaptide also appear to activate both GPR37 and GPR37L1. However, these pairings are yet to be universally acknowledged. Both receptors are widely expressed in the brain. GPR37 has been implicated in Parkinson's disease and parkinsonism, while GPR37L1 deletion leads to precocious cerebellar development and hypertension.

Stockert and Devi review the recent progress in GPCR structure determination and structure-based virtual screening for identifying ligands for orphan receptors. Advances in protein crystallography have led to the determination of over 120 X-ray structures of GPCRs, some of which are ligand bound. These structures can serve as templates to develop homology models of orphan GPCRs, which, in turn, can be used to virtually screen ligands for these receptors. Success has been made to discover novel potent ligands for the H_4_ receptor, FFAR1, and several other orphan GPCRs. The structure based approach holds great promise in drug discovery targeting orphan GPCRs.

Altogether, the topic covers the molecular pharmacology, biology, and potential clinical applications of several orphan GPCRs including FFARs, H_4_ receptor, EIB2, GPR35, GPR55, GPR37, and GPR37L1. In addition, structure-based virtual screening emerged as an exciting approach to facilitate drug discovery for orphan GPCRs.

## Author contributions

YF, TK, and CL wrote the manuscript.

### Conflict of interest statement

The authors declare that the research was conducted in the absence of any commercial or financial relationships that could be construed as a potential conflict of interest.

